# Microscopic Illustrations of Living Objects

**Published:** 1840-10

**Authors:** 


Art. VI.—*Microscopic Illustrations of Living Objects; with Researches concerning the Construction of Microscopes and Instructions for using them.* By C. R. Goring, m.d. *A New Edition, emended and enlarged.* By A. Pritchard, m.r.i. &c.—*London,* 1840. 8vo. pp. 248.

Now that microscopic investigations are obtaining so much attention by the profession, we are glad to observe that the above interesting and important work, although increased considerably in size, has been reduced to less than one half its original price. The work is a strictly practical one, written by practical men, and the present edition brings the subject down to the present time. We recommend it to the attention of our readers.

